# Granular Cell Tumor of the Colon: A Rare Diagnosis in an Uncommon Location

**DOI:** 10.7759/cureus.85226

**Published:** 2025-06-02

**Authors:** Saar Peles, Alik Manoogian, Michael Roth, Zachary Field

**Affiliations:** 1 Department of Gastrointestinal Oncology, University of Central Florida College of Medicine, Orlando, USA; 2 Department of Internal Medicine, University of Central Florida College of Medicine, Orlando, USA; 3 Department of Gastroenterology, Digestive Disease Consultants, Orlando, USA

**Keywords:** colon cancer and colon polyps, colonoscopy, granular cell, rare cancers, schwann cell neoplasm

## Abstract

Granular cell tumors (GCTs) are rare neoplasms of Schwann cell origin that typically exhibit benign behavior but can rarely undergo malignant transformation. Gastrointestinal involvement is uncommon, and colonic localization is particularly rare. We report the case of a 51-year-old African American woman referred for evaluation of iron-deficiency anemia. Colonoscopy revealed a 10-mm submucosal lesion at the hepatic flexure with a characteristic yellow-white appearance. Histopathologic examination showed polygonal cells with abundant granular eosinophilic cytoplasm and small, uniform nuclei. Immunohistochemical staining was strongly positive for S100, SOX10, and inhibin, confirming the diagnosis of a GCT. There was no evidence of necrosis or mitotic activity. Although the lesion lacked high-risk histologic features, given the tumor’s submucosal origin and its uncertain malignant potential, future endoscopic resection will be pursued. This case demonstrates the importance of considering GCTs in the differential diagnosis of submucosal colonic lesions and highlights the diagnostic value of immunohistochemistry. It also emphasizes the need for surveillance in populations potentially at higher risk of malignancy, even in the absence of overt histologic features.

## Introduction

Granular cell tumors (GCTs) are rare neoplasms of Schwann cell origin, characterized by polygonal cells with eosinophilic, granular cytoplasm on electron microscopy and diffuse S100 positivity on immunohistochemistry [1]. While the majority of GCTs are benign, malignant transformation occurs in approximately 1%-2% of cases [2]. These tumors can arise in various anatomical sites, though their occurrence in the gastrointestinal (GI) tract is uncommon. Within the GI tract, they most commonly occur in the esophagus, followed by the stomach and duodenum [1]. Colonic GCTs are exceedingly rare and are most commonly detected incidentally during routine colonoscopy or other screenings [1]. Here we present a rare case of a colonic GCT.

## Case presentation

**Figure 1:**
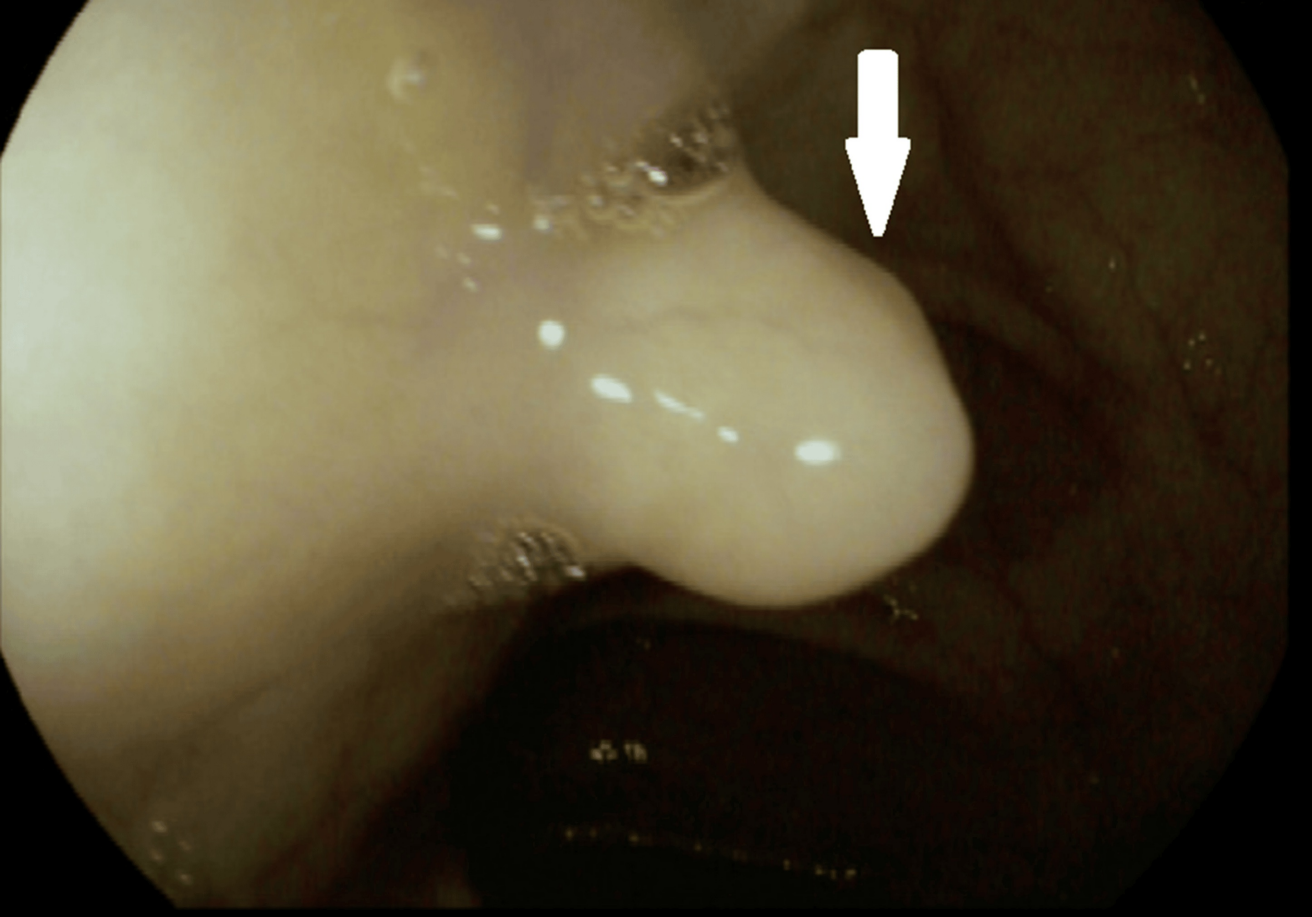
Granular cell tumor seen during colonoscopy

**Figure 2:**
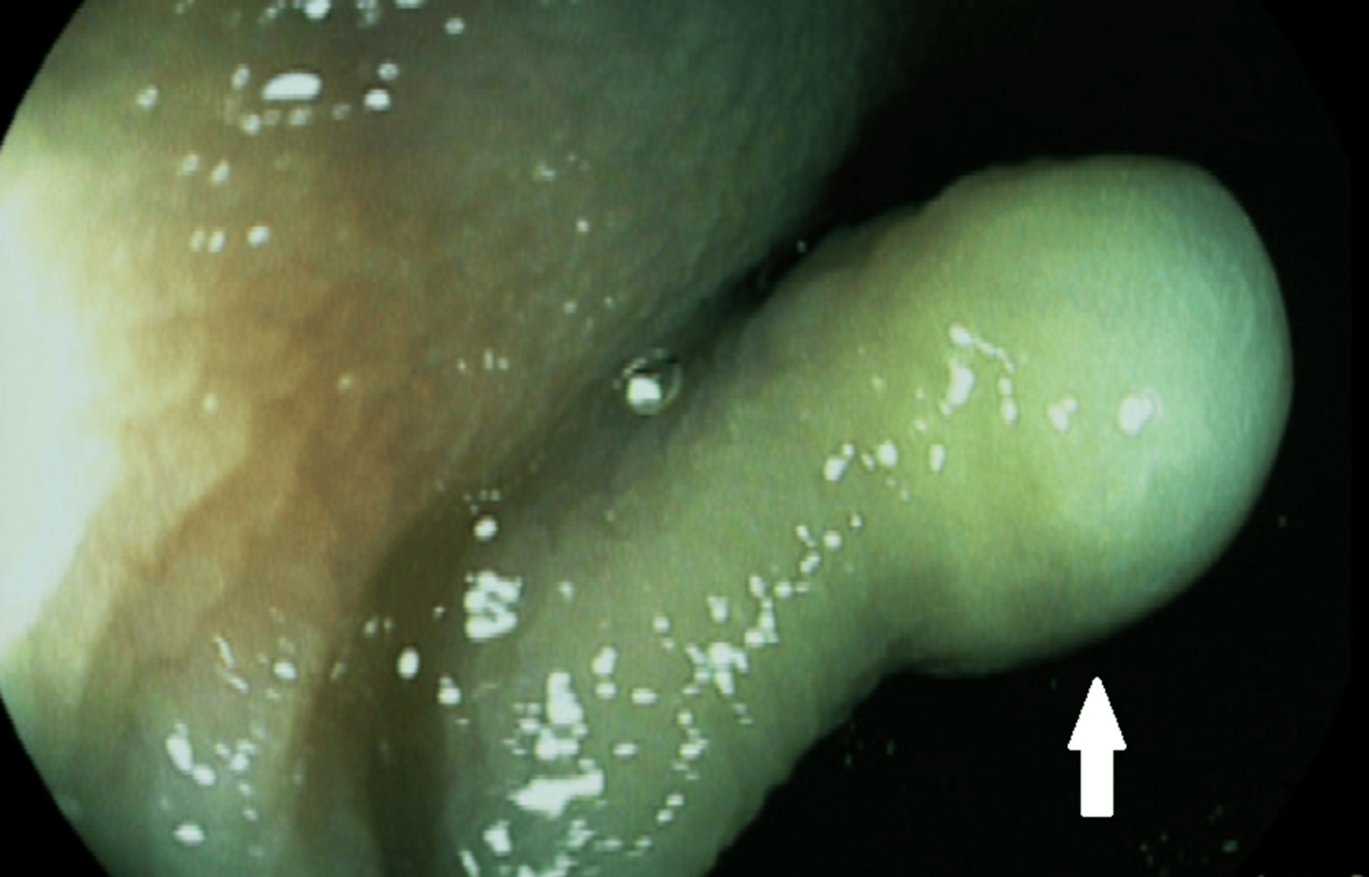
Granular cell tumor under i-SCAN

A 51-year-old African American woman with a previous history of menorrhagia presented to a gastroenterology clinic for referral by her hematologist for management and evaluation of iron-deficiency anemia. The patient underwent an esophagogastroduodenoscopy (EGD) and an index colonoscopy, with the EGD notable for mildly atrophic gastric mucosa and an otherwise normal examination. Gastric biopsies were positive for *Helicobacter pylori* gastritis, and duodenal biopsies were normal. Colonoscopy noted normal terminal ileum, a 5-mm nodule in the cecum (biopsies with submucosal adipose tissue suggestive of submucosal lipoma), a 10-mm nodule at the hepatic flexure (biopsied with cold forceps, indicating GCT in Figures 1, 2), two sessile polyps in the sigmoid colon (hyperplastic polyps), one 6-mm polyp in the rectum (tubular adenoma), and nonbleeding internal hemorrhoids. As shown in Figure 2, the mass did not have any pit patterns under i-SCAN (Pentax Medical, Tokyo, Japan), a software that allows modifications of sharpness, hue, and contrast to enhance mucosal imaging, used in endoscopic studies, indicating that it was not a polyp.

Immunohistochemical staining was performed to further characterize this cellular proliferation. Discovered on GI stromal tumor 1 was negative, ruling out a GI stromal tumor. Smooth muscle actin was also negative, excluding a leiomyoma. CD68 was negative as well. S100, Sox10, and inhibin immunohistochemical stains were strongly and diffusely positive within the polygonal granular cells, and a diagnosis of GCT was made. No mitotic figures were identified, and there was no necrosis present. While there were no high-risk features present, suggestive of malignancy, because of the diagnostic uncertainty, and as malignant cases appear to be more prevalent in the patient’s demographic of African-American women, the patient and physician did elect to remove the tumor endoscopically.

She was successfully treated with bismuth quadruple therapy and is continuing with oral iron replacement, with plans for endoscopic resection in the near future.

## Discussion

GCTs are soft tissue neoplasms thought to arise from Schwann cells and can be found in any part of the body. They most often follow a benign course but may rarely become malignant (1%-2%). They have an abundant eosinophilic cytoplasm and are S100 positive due to their neural origin [1].

Designating GCTs as benign or malignant presents many diagnostic challenges for both gastroenterologists and pathologists. While pathologists may generally categorize GCTs as benign, in daily practice, this becomes more complicated, as GCTs have malignant potential [3]. While tumors with metastasis can be easily classified as malignant, malignancy can be identified without metastases, similarly to other soft tissue tumors. As malignant GCTs are not well understood, this presents challenges in daily practice for managing such tumors.

Fanburg-Smith et al. proposed classifying GCTs based on six histopathological criteria to prognosticate and identify tumors with potentially more aggressive behavior [4]. The six criteria are necrosis, spindling, vesicular nuclei with large nucleoli, increased mitotic activity (>2 mitoses/10 high-power fields at 200× magnification), high nuclear to cytoplasmic (N:C) ratio, and pleomorphism. Neoplasms that display only focal pleomorphism are considered benign; neoplasms that meet one or two criteria are classified as atypical; neoplasms that meet three or more criteria are considered histologically malignant [4]. Some of the proposed criteria, including pleomorphism and an elevated nuclear-to-cytoplasmic ratio, are prone to interobserver variability and demonstrate limited reproducibility among pathologists. This inconsistency can complicate the diagnostic process and blur the distinctions between various diagnostic subgroups. As a result, Nasser et al. attempted to refine the classification criteria based on just two criteria: the presence of necrosis and/or mitosis [5]. While neither of these classification systems has been well validated on a large scale, likely due to the rarity of GCTs, they can be used to prognosticate tumors into lower and higher risk. This is critical, as malignant GCTs have a 60% survival rate within three years, with most cases reported in African-American women [6].

Smaller GCTs have been successfully resected en bloc using endoscopic submucosal resection (ESD) and endoscopic mucosal resection (EMR) [7]. However, endoscopists must keep in mind that, as the name in EMR implies, this technique resects the mucosa, while GCTs originate from the submucosa. As a result, ESD may be a more effective strategy to ensure complete tumor resection. For larger tumors (3-5 cm), endoscopic submucosal excavation has also been used successfully.

## Conclusions

GCTs of the colon are an exceptionally rare finding on colonoscopy and often present as small, yellow-white, well-circumscribed, mucosa-covered masses. They are typically benign and asymptomatic but may present with bowel habit changes, hematochezia, or abdominal discomfort. On pathology, they are characterized by polygonal cells with eosinophilic, granular cytoplasm on electron microscopy and diffuse S100 positivity on immunohistochemistry.
